# Practical Remarks on Congestive Fever

**Published:** 1846-12

**Authors:** E. F. Bouchelle

**Affiliations:** Columbus, Mississippi


7. *Practical Remarks on Congestive Fever.* By E. F. Bouchelle, M. D., of Columbus, Mississippi.—In perusing the last edition of Stokes and Bell's Practice of Physic—a work embodying many valuable principles of medicine with great experience and learning—I am forcibly impressed with the views of Dr. Bell, as almost coincident with my own, as it regards the efficacy of opium in the treatment of congestive fever. I have long been satisfied in my own mind, that the usual mode of treating congestive fever, the plan pursued by most of the physicians of the south west, is not only improper, but dangerous; as its direct tendency is to strengthen the disease, and hasten the stage of collapse. The views which I now entertain on the subject of congestive fever have been promulgated throughout the sphere of my acquaintance, since the summer of 1837.

It is perhaps unnecessary to advance, in detail, a theory of the disease in question; suffice, for all practical purposes, to remark, that all of the leading phenomena of the disease are referrible to derangement of the organic system of nerves, more particularly. The excitement of congestive fever is irritable excitement, and in most cases so excessive, that it soon sinks the system into collapse unless moderated.

Such being a *syllabus* of my pathology, it necessarily follows that in its treatment I invariably call in requisition those remedies whose known tendency is to allay nervous irritation, tranquillize the system, and produce sleep. Such remedies are to be found under the class of *narcotics*, and in another *great remedy* belonging to no particular class, which the hand of a merciful and all-wise Providence has disseminated throughout the universe; a remedy equally accessible to the rich man and the poor man, as it abounds in all places, and can be procured "without money and without price." I allude to *cold water*. The most powerful combination, however, to prevent the recurrence of a paroxysm, when the disease observes a remittent or an intermittent character, is morphine and quinine. In the whole course of my observation I have never known the congestive fever to observe any other than the intermittent or remittent type; unless the constitution is so frail, or the disease so violent, as to destroy the patient in the first paroxysm, which it often does; moreover, it is a rare circumstance if an individual, with the most robust constitution, survives a second or third paroxysm. Most usually, during the paroxysm, I prescribe *laudanum* and cold water, which rarely fail to conduct the patient safely through; and during the interval, morphine and quinine, to prevent a recurrence. The following is the prescription usually observed:—℞ Sulph. quinine, grs. xxiv.; Sulph. morphine, grs. ij.; M. f. 12 pills,—to take one sufficiently often to keep up a slight state of stupor or narcotism; that is, every hour or two, *pro re natâ.*

I am unfriendly to large doses of quinine, and am certain that two or three grain doses repeated at intervals, will insure all the good effects of that potent salt, without incurring the risk of losing them; not only losing, but inflicting an injury to the nervous system. Our firm belief is, and that opinion is founded on experience, that, as an antiperiodic, two grain doses of quinine are as efficacious as larger doses; and that in the same proportion as we augment the dose, in the same or a greater degree do we diminish the specific action of the article; also, that its combination with a narcotic enhances its antiperiodic powers in an eminent degree.

There is a secret in connection with quinine which, probably, very few physicians have observed; that is, that its administration during the stage of excitement in fever is often hurtful, and at best uncertain; in order to insure a favorable influence in such cases, we have only to combine it with an anodyne. It is rare that quinine will exert any other than a favorable influence during the hot stage of fever, provided morphine be blended with it. Its most common effect, under such circumstances, is to lessen the force and frequency of the pulse, relax the skin, and produce sleep. The above combination is an admirable prescription in the summer fevers attended with great gastric irritability,—it must be given in the form of pills. Another valuable combination, where the excitement is inordinate, is quinine, tartar emetic, and morphine,—provided there is no great nausea. The above refers only to summer and autumnal fevers, of open excitement.

Before leaving this subject, I will remark that 20, 60, and 100 grain doses of quinine are very common these days. However, such doses are not prescribed, or if so, by *very few* of the scientific physicians of Mississippi and Alabama. In the meanwhile I will not presume to deny that peculiar modifications of disease may render *such doses* applicable in more southern latitudes. Generally speaking, these huge doses are given by that numerous class of mountebanks and impostors who infest our country; men who recognize no essential difference between the stomach of a human being and that of an ostrich; between the constitution of a man and that of a horse! Would to God that the prescribing of large doses of quinine was the only species of quackery practised in the West! Calomel, and other remedies, are given in equally as large quantities; to the success of which *energetic empiricism*, our numerous grave-yards bear melancholy though silent testimony, to say nothing of the thousands of constitutions literally destroyed by as *many anomalous diseases!*

There is a maximum and a minimum dose for any article of the materia medica—a fact which should never be forgotten in clinical practice—and when we transcend either degree, we either produce no effect at all, or we do mischief.

There is no class of remedies, however, whose dose is more variable than that of narcotics. Indeed, we can sometimes give them *ad libitum*, with very little effect; as we all know, that under certain states of the nervous system arising from excessive pain, the system can scarcely be composed by opiates. Who has not seen this verified in prescribing for acute gout, the passage of biliary calculi, spasmodic cholic, tetanus, &c. &c.? One of these peculiar conditions of the system occurs in congestive fever,—as we are certain that during one of its paroxysms nothing short of mammoth doses will conduct the patient safely through, and prevent collapse; which extraordinary resistance to the usual influence of opiates only argues the propriety and necessity of such remedies. I do hope, for the sake of human life, and the honor of medicine, that the day will, ere long, arrive when physicians will be convinced that calomel and purgatives generally, French brandy and other stimulants, mustard cataplasms, blistering plasters, &c., are not the remedies for congestive fever, the endemic of the Mississippi valley, whose very name, in many places, is associated with all the horrors of the grave, in consequence of its great fatality. All purgatives, all stimulants, internal or external; all irritants—are injurious in congestive fever. So long as I pursued the plan of *correcting the secretions, and stimulating by brandy, camphor, camphor and quinine, ammonia, pepper, &c.*, I lost patients. But when, on the other hand, after much reflection, I had changed my pathology of the disease, and adopted the *cold water* and *anodyne* practice, my labors were crowned with success, and have been ever since. In truth, the most violent forms of congestive fever will as certainly yield to the anodyne treatment, as will a local inflammation yield under depletion. I do not regard quinine as a stimulant; it has tonic properties, and in combination with an anodyne, is the most powerful sedative in general use. (There are many sedatives very active, which are not used in the common routine of clinical practice.)

We have said nothing definite, as yet, about cold water in congestive fever, but will do so in very few words. How is the cold water used in congestive fever? Internally and externally; a pleasant remedy, and one which any patient will grasp eagerly, and without much persuasion. I use the cold douche in collapse to arouse the system to reaction, which it will more often do, than any other means that I have ever seen essayed. I have seen many patients, as it were, moribund; cold and clammy skin, thready pulse, sunken features, blue finger nails and lips, great epigastric oppression, and breathlessness, rescued, as it were, from the grave, by the *magic influence* of the cold douche. The cold water is not less useful during the paroxysm, to allay general anxiety, distressing vomiting, thirst, and internal heat. I allow the patient to drink it freely,—it gives great relief; it removes, in connection with laudanum, irritation of the ganglionic nerves, upon which the miserable epigastric oppression and gastric irritability depend, and seldom fails to conduct the patient safely through the paroxysm. How much more rational such treatment is, and, at the same time, how much more grateful to the languishing sick man, than the opposite plan of tormenting him *unto death* with heating stimulants and *blistering plasters!* How much more rational than the opposite *vile* system of cramming his stomach with horse-doses of calomel "to remove congestion" of the *darkest* and *foulest* of all places, "*the venous cavity*"*!!!* Would to God that Mississippi and Alabama could be relieved of the curse of R. A. C. quackery! Oh! ye shades of departed worth! ye ghosts of Hippocrates, Æsculapius, and Galen, how long will ye endure such humbuggery! Oh!

"*venous cavity*"*!* Oh! calomel, and R. C. A. pills! inexorable monsters, who have slain your hundreds, why seek to demolish thousands! I am not jesting; no, I am serious.*

But, for the purpose of illustrating the most rational practice in congestive fever, I will submit one of the most violent cases I ever saw in Mississippi.

*Case.*—A particular friend, of vigorous constitution, was seized about midnight on the 20th of September, 1845, with a slight chill, which was succeeded by vomiting, and profuse liquid evacuations from the bowels. I saw the patient about 9 on the 21st; his head was hangiug over the side of the bed, and he incessantly vomiting or heaving; his features were sunken and pale; breathing rapid, and difficult from congestion of the lungs; pulse feeble and very rapid, almost imperceptible at the extremities; lips blue, tongue pale and moist; with a clammy exudation of viscid perspiration all over the surface. Indeed I was surprised to find my friend on the very verge of the grave: that he was sinknig rapidly into a deadly collapse. He complained of great thirst and universal heat; he would cry out, "My God, I must have fresh air, or I'll die; I am burning up!" when the pulse was gone at the extremities, and the skin cold. The friends around implored me to stimulate him, and apply sinapisms to the extremities. I refused, and immediately went to work in my own way. I gave him 100 drops of laudanum forthwith, and in half an hour gave 50 drops more, which he drank. Seeing that the irritability of the system was so excessive, that the laudanum would not take effect, unless repeated at short intervals—in half an hour more I gave him 100 drops by enema. In an hour the vomiting stopped—my friend drinking cold water by the pitcher full. He very soon became tranquil, and fell into a deep sleep, with his mouth and eyes half closed. The spectators around thought that he was dying; but I knew better, when I took hold of his hand and found that it was getting warm, and that the pulse was rising at the wrist. In the course of two hours more, my patient was under a full re-action; his skin warm and pulse full, beating eighty in the minute. He did not wake until sundown—when he *got up*, dressed himself, and *went about his usual business!!*

The next morning (22d) I entreated him to take to his bed, and commence with the quinine and morphine, to prevent a recurrence of the paroxysm, which would take place about midnight.—He declined, stating that he was well. However the poor fellow was seized again at one o'clock on the 23d. In two hours he was vomiting forcibly, with frequent liquid dejections from the bowels; great dyspnœa, and small and

* I do not allude to Prof. Cooke; but to those who endeavor to treat the fevers of Mississippi, Alabama, &c., according to his theory. I respect the Professor, at the same time I am convinced of his delusion.

rapid pulse, with cold skin. At daylight I saw him, and gave the first dose, which was 100 drops of laudanum. Seeing that he became worse, complaining of indescribable epigastric heat and oppression, I repeated the dose, which had no effect, as he soon became wild and unmanageable. I ordered 100 drops more by enema, in starch; at the same time allowing him to drink freely of cold water, acidulated with citric acid, which he drank, in his derangement, with all the avidity of a famishing animal. He soon became cold from head to foot; *no pulse*, skin cold and bathed in a viscid sweat, lips blue, eyes sunken, and features shrivelled; breathing slow, and oppressed from congestion of the lungs. Indeed, the dyspnœa was so great, that he looked very much like a man suffocating. I ordered 100 drops more in enema, and applied two small sinapisms to the neck, one over each pneumogastric nerve, recollecting to have read of such things being useful in asphyxia, &c. In a short time the patient seemed more quiet,—drinking freely of cold water occasionally. At this juncture, a medical friend of experience, formerly of the United States Navy, stepped in and pronounced my patient *in articulo mortis;* however, before he had been present one hour, the pulse was rising at the wrist, and the skin began to get warm, and the patient to breathe with more ease. In two hours more my patient was lying in a profound sleep, with hot skin and good pulse; with the warm sweat standing in great drops on his forehead. He awoke late in the evening, very much prostrated indeed. In a short time I put him under the morphine and quinine, keeping up slight narcotism until the next period had passed in safety; when I gave a little blue pill occasionally, to restore the secretions. It is proper to mention here, that the use of laudanum and quinine, as above recommended, almost always leaves the system in a torpid condition, as manifested by a coated and dry tongue; so that convalescence will be tedious without the occasional use of a little blue pill, &c.

The patient whose case I have given, cannot bear the smallest quantity of laudanum when well. I could, if necessary, adduce other cases, showing conclusively that laudanum, cold water, and quinine, are the remedies for congestive fever. It is probable that the above patient would have died, had it not been for the plasters (size of a dollar) to the neck; or it may be that the laudanum had not taken effect until then.

The treatment which has just been detailed in a detached and hurried manner, with some little modification, is applicable to any form of summer and autumnal fever in Mississippi. There is no prescription better, in common evers, to prepare the system for quinine, than morphine and tartar emetic in solution. Ordinary febrile excitement can resist its influence but a few hours. In conclusion.—There is no class of remedies which exert so favorable an influence in all of the fevers of this latitude, as the class of narcotics.—*West. Lan.* in *Bull. of Med. Science.*

